# Solutions for maximum coupling in multiferroic magnetoelectric composites by material design

**DOI:** 10.1038/s41598-018-22964-9

**Published:** 2018-03-20

**Authors:** K. P. Jayachandran, J. M. Guedes, H. C. Rodrigues

**Affiliations:** IDMEC, Instituto Superior Técnico, University of Lisbon, Av. Rovisco Pais, 1049-001 Lisbon, Portugal

## Abstract

Electrical control of magnetization offers an extra degree of freedom in materials possessing both electric and magnetic dipole moments. A stochastic optimization combined with homogenization is applied for the solution for maximum magnetoelectric (ME) coupling coefficient *α* of a laminar ME composite with the thickness and orientation of ferroelectric phase as design variables. Simulated annealing with a generalized Monte Carlo scheme is used for optimization problem. Optimal microstructure with single and poly-crystalline configurations that enhances the overall *α* is identified. It is found that juxtaposing a preferentially oriented ferroelectric material with a ferromagnetic ferrite into a composite would result in manifold increase in magnetoelectric coupling. The interface shear strains are found to be richly contributing to the ME coupling. The preferential orientation of the ferroelectric phase in the optimal ME composite laminate is demonstrated using the optimal pole figure analyses.

## Introduction

Magnetoelectric (ME) multiferroics have recently drawn increasing interest due to their potential applications in multifunctional devices such as nonvolatile memory elements, nano-electronics, etc.^1,2^. Natural multiferroic single-phase compounds are rare and their ME coupling responses are either weak or occurs at very low temperatures^1,3^. Nevertheless, substantial ME effect can be derived through fabricating composites of a ferroelectric (FE) and a ferromagnetic (FM) material in the form of thin film multilayered heterostructures, laminates, particulate or fiber–matrix composites etc^1,4^. Selecting materials possessing highly contrasting lattice parameters (thereby ensuring spontaneous immiscibility of phases) such as the perovskite-spinel system of electrostrictive and magnetostrictive phases in a laminar composite architecture could yield better ME coupling than single-phase materials or particulate composites^1^. Various efforts to improve the value of ME coupling coefficient *α* have been made by modifying preparation techniques of the samples, by the proper choice of materials or different structures, or by choosing different thickness of the samples^5,6^.

Theoretical characterization of equivalent material properties of complex material systems such as multiferroics is pursued as an alternative to experiments which are hampered by a host of factors affecting the sample such as demagnetization, debonding, microcracks, interdiffusion, depoling etc.^7–10^. The idea behind the development of ME multiferroic composites is to generate the desired magneto-electric effect as a strain induced *product property*^11,12^. The *product property* in a composite is defined as an effective property which is not present in either of the constituent phases^13^. Several analytical and computational models have been developed to quantify the product property of magnetoelectric coupling in ME multiferroic composites^8,12–16^ consequent to the strain-mediated two-phase model proposed by Harshe *et al*.^4,17^. Green’s function technique was developed by Nan^18^ for the solution of the constitutive equations and thereby compute the effective properties of ME composites. Micromechanics models too were developed as an alternative method to accomplish the same^19–21^. Theory of low-frequency magnetoelectric coupling in magnetostrictive-piezoelectric bilayers was developed by Bichurin *et al*.^15^. The ME equivalent circuit and corresponding ME coefficients have been derived for laminates by Dong *et al*.^22^. Phase-field models have been developed for predicting and interpreting both the equilibrium domain structures and domain switching kinetics by simulating the mesoscale microstructure of the magnetoelectric materials^23^. Nonetheless, most of the theoretical developments confine to two-phase materials which constitutes a subset of the multiferroics. The homogenization framework used in this paper is not specifically limited to two-phase composites alone, but provides a general platform to treat the equivalent (linear) static coupling interactions of all forms of multiferroics *viz*. single crystals, polycrystals, as well as all kinds of multiferroic composites irrespective of the crystallographic symmetry of the materials. The two-scale asymptotic analysis combined with a variational formulation of the underlying electrical, mechanical and magnetic fields would unveil their interaction in the microscopic scale.

Large ME coupling is indispensable for practical applications^1,8^. Recently multiferroic laminate composites of the transversely poled Poly(vinylidene fluoride–co–hexafluoropropylene-P(VDF-HFP) and the iron-based Metglas^24^ was found to show ME voltage coupling *α*_*E*33_ as high as 320 V/cmOe. Large ME effects in a host of composites, including thin film heterostructures on nickel zinc ferrite, thick films of nickel ferrite and a piezoelectric deposited by aerosol deposition, in multiferroic composite bimorph structures are reported recently^9,25–27^. Recent advances in manufacturing single crystal oriented multilayer films through heteroepitaxial growth and multilayering are found to enhance the magnetoelectric coupling^6^. If large ME coupling is achieved by upscaling the existing materials and geometries, it would cater to the economy of resources. We model the optimal material design ideal to accomplish this goal by combining the homogenization procedure with a stochastic optimization method.

In this paper, we are optimizing the microstructure (MS) of a laminar ME composite of two ferroic materials (ferromagnetic CoFe_2_O_4_ (CFO) and ferroelectric BaTiO_3_ (BTO)) that possessed contrasting lattice parameters^28^. To maximize the ME coupling, we search -by employing the method of simulated annealing based in the Metropolis algorithm- for the optimal MS configuration that could enhance this effect especially utilizing the inherent anisotropy of the component phases in composite multiferroics^29,30^. Recent advances in manufacturing oriented single crystal multilayer films through heteroepitaxial growth on top of a crystalline substrate offer grounds for such a study as this could be realized into potential device applications^1,6^. As a guide to the experimental realization of the ME composite, we explore the texture information through the optimal pole figure analysis.

Problems inherent to bulk ME composites such as destruction of piezoelectric charges by leakage current can be avoided in horizontal layered geometries^1,15^. Since the lattice mismatch between CFO and BTO is large (~0.52), it can either give rise to an heteroepitaxial strain in ME thinfilms^31,32^ or enhanced interface stress in other horizontal layered media^4,28^. The ME coupling coefficient tensor *α*_*ij*_ corresponds to induction of electric polarization by a magnetic field or of magnetization by an electric field and is designated as the linear ME effect^7^. *α* of a laminar ME composite is inextricably related to the thickness and orientation (or design variables) of FE phase. As *α* could not be expressed explicitly as function of the design variables, the optimization problem should be treated as a combinatorial optimization with a generalized Monte Carlo scheme^30^. A three-dimensional (3D) generic model is developed to compute the homogenized ME property tensor of a multiferroic possessing the least crystallographic symmetry (i.e., triclinic) to be interfaced with the optimization program^33^. See Supplementary Material for further details.

## Optimization of magnetoelectric composite

The general homogenization method applied to magnetoelectric composite is based upon assumptions of periodic boundary conditions on the microstructure and the separation of the microstructure scale through asymptotic expansion. The asymptotic analysis of multiferroic ME material leads to the product property of the homogenized ME coupling^33^

1 $$\begin{array}{rcl}{\tilde{\alpha }}_{ij} & = & \frac{1}{|Y|}\,{\int }_{Y}\,[{e}_{pkl}({\bf{x}},{\bf{y}})\frac{\partial {{\rm{\Gamma }}}_{k}^{m}}{\partial {y}_{l}}-{\kappa }_{pj}^{\epsilon H}({\bf{x}},{\bf{y}})\frac{\partial {{\rm{\Psi }}}^{m}}{\partial {y}_{j}}\\  &  & -{\alpha }_{pj}({\bf{x}},{\bf{y}})\,({\delta }_{jm}+\frac{\partial {Q}^{m}}{\partial {y}_{j}})]\\  &  & \times ({\delta }_{np}+\frac{(\partial {R}^{n})}{\partial {y}_{p}})\,{\rm{d}}Y\end{array}$$

Here *R* and *Q* are characteristic electric and magnetic displacements. **Γ**, and Ψ are characteristic coupled functions satisfying a set of microscopic equations^33^. These characteristic functions appear in the microscopic displacement, electric and magnetic field perturbations, *viz*., **u**^**1**^(**x**, **y**), *φ*^1^(**x**, **y**) and *ψ*^1^(**x**, **y**) respectively, due to the microstructure heterogeneity. The spacial derivatives of the macroscopic displacement, electric and magnetic fields *viz*., **u**^**0**^(**x**, **y**), *φ*^0^(**x**, **y**) and *ψ*^0^(**x**, **y**) can bridge the macroscale and the microscale through the characteristic functions mentioned above. *e*_*pkl*_ and ${\kappa }_{pj}^{\epsilon H}$ are the piezoelectric coefficient and dielectric permittivity at constant strain $\epsilon$ and magnetic field vector **H** respectively. $i,j,k,l,\cdots =1,2,3$ are the 3–dimensional coordinate indices, and *δ*_*ij*_ are the Kronecker delta symbol. Here we assume Einstein convention on summation about repeated indices. (Here the ~ over Greek or Latin letters represents homogenized values). We consider the ME composite as a heterogeneous body obtained by the translation of microstructure of size *Y*. For the homogenized ME composite laminate, the physical properties do not depend on **x**, the global frame of reference. Instead, if the material is heterogeneous, the magneto-electro-mechanical properties effectively depend on **x** such that ${\kappa }^{\epsilon H}\equiv {\kappa }^{\epsilon H}({\bf{x}}),\,{\bf{e}}\equiv {\bf{e}}({\bf{x}}),\ldots$ etc. The material properties ${\kappa }^{\epsilon H}$, **e**, *α*, etc as well as the characteristic functions *R*, *Q*, **Γ**, and Ψ are *Y*–periodic functions depending on the microscopic coordinates **y**.

Since the homogenized or effective $\tilde{\alpha }$ is the measure of the efficiency of the control of the electrical voltage on the magnetization state of the ME composite, its maximization would be the key objective sought in potential applications^34^. It is possible to obtain other relevant and experimentally measured ME parameters such as the effective ME voltage coefficient $\tilde{{\alpha }_{E}}=\delta E/\delta H$ through the knowledge of $\tilde{\alpha }$ in such a way that $\tilde{\alpha }=\delta P/\delta H=\tilde{{\alpha }_{E}}{\kappa }_{0}\tilde{\kappa }$ of the composite. Here, E, P and *κ*_0_ are the electric filed, polarization and permittivity of free space respectively. $\tilde{\kappa }$ is the effective relative permittivity of the composite.

### Single crystal BTO–ceramic CFO

The homogenized ${\tilde{\alpha }}_{33}$ and ${\tilde{\alpha }}_{11}$ are set as the objective functions in this study. (Here the ~ over *α* represents homogenized values). ${\tilde{\alpha }}_{33}$ and ${\tilde{\alpha }}_{11}$ depends on the detailed configuration (anisotropy characterized by the orientation of the constituent phases and volume fraction or thickness of the constituent phases) of that system (here the ME composite laminate). Using the objective function ${\tilde{\alpha }}_{ij}({\bf{k}})$ and defining configurations by a set of parameters {**k**}, it is straightforward with the Metropolis procedure to generate a set of configurations of a given optimization problem at some effective *temperature* (a control parameter)^30^. See Supplementary Material for further details. In single crystalline BTO-ceramic CFO laminate the optimization problem is to find vector {**k**} of design variables to minimize the objective function ${\tilde{\alpha }}_{ij}$, i.e.,

2 $$\begin{array}{l}{\rm{find}}\quad {\bf{k}},\quad {\rm{\min }}\quad {\tilde{\alpha }}_{ij}({\bf{k}})\\ {\rm{where}}\quad {\bf{k}}=(\varphi ,\theta ,\psi ,{v}_{f})\quad {\rm{of}}\,{\rm{BTO}}\,{\rm{layer}}\\ {\rm{s}}.{\rm{t}}\mathrm{.,}\quad 0 < {v}_{f} < 1\quad {\rm{and}}\\ {\rm{s}}.{\rm{t}}\mathrm{.,}\quad -\pi \le (\varphi ,\theta ,\psi )\le \pi \end{array}\}$$

**Figure 1:**
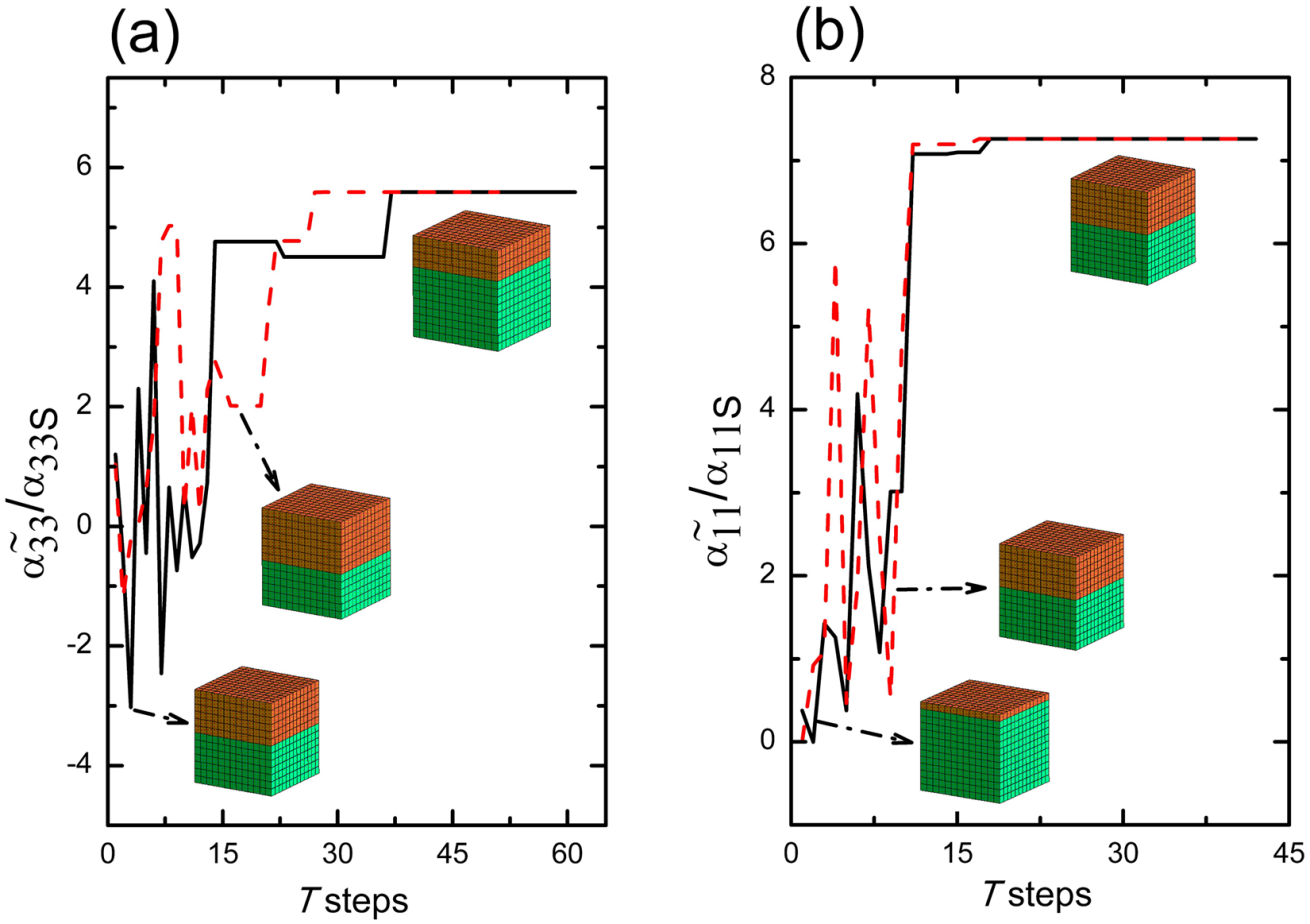
Convergence of the normalized ME coupling coefficients, (**a**) ${\tilde{\alpha }}_{33}$ and (**b**) ${\tilde{\alpha }}_{11}$ vs *temperature* steps of the single crystal BTO–ceramic CFO laminate for two initial guesses each. The MSs of BTO (in green)-CFO (in brick red) shown (at the bottom) inset corresponds to the initial guesses and the final solution (top ones).

Two problems viz., the optimization of ${\tilde{\alpha }}_{33}$ and ${\tilde{\alpha }}_{11}$ are summarised in the above Eq. 2. Here, the orientation of the single crystal BTO layer of ME composite laminate possessing a volume fraction *v*_*f*_ is characterized by the Euler angles (*ϕ*, *θ*, *ψ*). We first optimize a laminate with the objective of maximizing the $|{\tilde{\alpha }}_{33}|$. The minimization program depicted in Eq. (2) should manifest the real optimization problem where the objective functions ${\tilde{\alpha }}_{33}$ or ${\tilde{\alpha }}_{11}$ conventionally assume negative values^33^. Multiferroic brick elements possessing five degrees of freedom (DOF) *viz*., three for displacements, one each for the electric and magnetic potentials and a total of 13, 720 DOF are used for the computation of homogenized ${\tilde{\alpha }}_{ij}$. See the Supplementary material for further details. The results of two trials of the normalized ${\tilde{\alpha }}_{33}$ and ${\tilde{\alpha }}_{11}$ are shown in Fig. 1(a,b). (Here the “step length” or the number of iterations in each step is set to be four.) Here the ${\tilde{\alpha }}_{ij}$ of the optimized system is compared against the bulk ${\tilde{\alpha }}_{ijS}$ values previously computed for isotropic composite. The inset to Fig. 1 depicts the MS with constituent volumes alone, while the optimal orientations of the FE phase are given in Table 1.

**Table 1:** The normalized optimal values of effective ${\tilde{\alpha }}_{11}$ and ${\tilde{\alpha }}_{33}$ of the single crystal BTO – ceramic CFO composite laminate and the corresponding solutions v_*f*_ and (*θ*, *ϕ*, *ψ*) (in degrees) of the FE component.

Objective $\widetilde{{{\boldsymbol{\alpha }}}_{{\boldsymbol{ij}}}}$	Case	Solution	*v*_*f*_	$\tilde{{{\boldsymbol{\alpha }}}_{{\boldsymbol{ij}}}}/\tilde{{{\boldsymbol{\alpha }}}_{{\boldsymbol{ijS}}}}$ (Normalized)
$\widetilde{{\alpha }_{33}}$	1	*ϕ* = 35, *θ* = 0, *ψ* = 85	0.69	5.59
	2	*ϕ* = 160, *θ* = 0, *ψ* = 170	0.69	5.59
	Experiment^a^	*ϕ* = arbitrary, *θ* = 0, *ψ* = arbitrary	0.78	—
	Experiment^b^	[100] BaTiO_3_/[100] CoFeO4	0.65	—
$\widetilde{{\alpha }_{11}}$	1	*ϕ* = −105, *θ* = 0, *ψ* = 155	0.54	7.26
	2	*ϕ* = 165, *θ* = 0, *ψ* = 160	0.54	7.26

^a^CoFe_2_O_4_-BaTiO_3_ heterostructured films epitaxially grown on the 001–SrTiO_3_ substrate via pulsed laser deposition possessing an inplane-substrate orientation relationship (001) BaTiO_3_‖(001) CoFe_2_O_4_‖(001)SrTiO_3_ from ref.^35^.

^b^Heteroepitaxial films of BaTiO_3_–CoFe_2_O_4_ grown on SrTiO_3_ substrate from ref.^36^. Here the orientation was measured to be [100] BaTiO_3_/[100] CoFe_2_O_4_ with respect to the substrate.

The solutions obtained show that the *c*–axis of the single crystal BTO lies parallel (*θ* = 0) to the *y*_3_ axis of the MS reference frame (*y*_1_, *y*_2_, *y*_3_) (Table 1). The *y*_3_ axis coincides with the spontaneous polarization direction. The optimal values for both ${\tilde{\alpha }}_{11}$ and ${\tilde{\alpha }}_{33}$ are found to be independent of the Euler angle *ϕ* which characterizes the *ab*-plane rotation of the BTO about the *y*_3_ axis (that coincides with the *c*-axis of the crystal before the Euler rotation is taking place). In this rotated geometry of the composite, the ME coupling attains manifold enhancements from the corresponding bulk composite values. Here we see the ${\tilde{\alpha }}_{11}$ attains more than 7 times the bulk value while ${\tilde{\alpha }}_{33}$ displays nearly 6-fold enhancement due to rotation of the FE phase. The optimal volume fraction *v*_*f*_ = 0.69 of single crystal BTO is in agreement with the experiments (Table 1) in BTO–CFO thinfilm^35,36^. Heteroepitaxial films of CoFe_2_O_4_ and BaTiO_3_ grown on (001)-SrTiO_3_ substrate exhibits the optimal *c*-domain orientation of BaTiO_3_ layer^35^. i.e., the c-axis of the BTO unit cell is parallel to the substrate normal or the Euler angle *θ* subtended by the c-axis would be *θ* = 0 as in our study. Yang *et al*.^37^ found that the *α* of the composite in a configuration with PFN–PT oriented with its [001] direction out of plane of the laminate is ~4 times as that with its [001] lying along the plane of the lamina. Our optimal *θ* value (*θ* = 0) in both instances of optimization (Table 1) shows that BTO oriented with its [001] axis out of plane of the lamina at maximum *α*.

ME effect in a composite is a coupled electrical and magnetic phenomena via elastic interaction^8^. i.e., for the magnetoelectric effect, when a magnetic field is applied to a composite, the magnetic phase changes its shape magnetostrictively. The strain is then passed along the piezoelectric phase, resulting in an electric polarization. Thus the strain-mediated ME effect in composites is extrinsic, depending on the composite microstructure and coupling interaction across magnetic-piezoelectric interface^38^. The property ME coupling *α* which is absent in the constituent phases thus originates in the composite. In order to explore the the enhancement of ME coupling at the optimal orientation, several factors were analyzed including interface strain field of the laminate. The laminate plane is essentially the xy-plane of the composite and the lamination is along the z-axis direction. Hence the interface is ideally spread along the xy-plane. The local distortion caused by the rotation of the BTO layer would impart additional strains at the interface besides the ones existing due to the lattice mismatch. We observe an additional component of elastic stiffness viz., *C*_16_ = 9.1 × 10^9^ *N*/*m*^2^ in the optimal composite in such a way that *C*_16_ = −*C*_26_ as shown in the elastic constant (*C*_*μν*_) matrix given below;

$$\begin{array}{rcl}(\begin{array}{cccccc}{C}_{11} & {C}_{12} & {C}_{13} &  &  & {C}_{16}\\  & {C}_{11} & {C}_{13} &  &  & -{C}_{16}\\  &  & {C}_{33} &  &  & \\  &  &  & {C}_{44} &  & \\  &  &  &  & {C}_{44} & \\  &  &  &  &  & {C}_{66}\end{array}) & = & (\begin{array}{cccccc}29.1 & 7.4 & 12.2 &  &  & 0.9\\  & 29.1 & 12.2 &  &  & -0.9\\  &  & 23 &  &  & \\  &  &  & 5.5 &  & \\  &  &  &  & 5.5 & \\  &  &  &  &  & 5.8\end{array})\\  &  & \times {10}^{10}\,N/{m}^{2}\end{array}$$

This property is non-existent in either of the BTO and CFO phases which were individually treated to be belonging to the 4 *mm* tetragonal class. Nevertheless, the *C*_*μν*_ matrix of the composite reveals that the overall symmetry of the optimal composite changed to one of the tetragonal classes $4,\overline{4},4/m$^39^. This symmetry change might be a consequence of the strain effect at the interface and this structural transition can bring about an enhancement of ME coupling *α* as it is seen here^40^.

**Figure 2:**
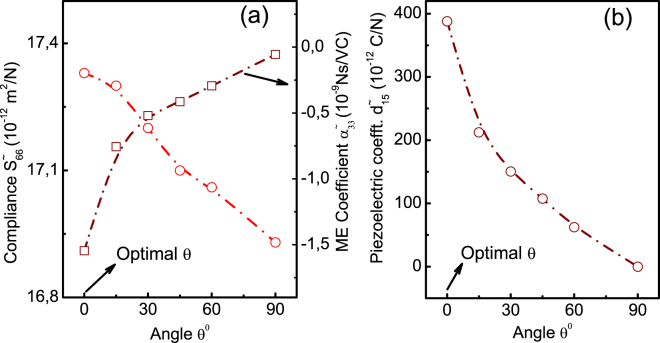
The variation of the homogenized (**a**) the elastic compliance *S*_66_ and (**b**) the piezoelectric strain coefficient *d*_15_ with the Euler angle *θ* of the single crystal BTO–ceramic CFO laminate.

The interface strain field developed as a result of the composite geometry and the rotation of the ferroelectric phase plays a crucial role in the magnetostriction in the ferromagnetic phase^41^. To further explore the interfacial strain effect on the ME coupling *α* we study the effective compliance ${\tilde{S}}_{66}$ which is equivalent to ${\tilde{S}}_{1212}\equiv {\tilde{\epsilon }}_{12}/{\tilde{\sigma }}_{12}$ in tensor form of the ME laminate at various orientations. Here ${\epsilon }_{12}$ and *σ*_12_ are the in-plane shear strain and stress respectively. Figure 2(a) depicts the effective compliance ${\tilde{S}}_{66}$ of the ME laminate and the corresponding out-of-plane ME coupling at various Euler angles *θ* which corresponds to the orientation of the *c*–axis of the BaTiO_3_ unit cell. It reveals that at optimal angle *θ* the ${\tilde{S}}_{66}$ attains the maximum (and so is the ME coupling $|{\tilde{\alpha }}_{33}|$) and decreases monotonically as we increase *θ*. The decreasing homogenized piezoelectric constant ${\tilde{d}}_{15}$ shown in Fig. 2(b) with *θ* reinforces the role of interfacial shear strain since ${\tilde{d}}_{15}\equiv {\tilde{d}}_{113}$ in tensor form. (*d*_113_ is essentially the piezostrain ${\epsilon }_{13}$ per unit electric field *E*_1_ acting along the x-direction of the crystal.). Thus the shear interfacial strains which get bigger at the optimal point in the solution space obviously contribute to the enhancement of ME coupling.

### Ceramic BTO–CFO composite

Next is the optimization problem of ceramic BTO–ceramic CFO laminate to find the vector **k** of design variables to minimize the objective function ${\tilde{\alpha }}_{ij}$ (i.e., either ${\tilde{\alpha }}_{33}$ or ${\tilde{\alpha }}_{11}$), i.e.,

3 $$\begin{array}{l}{\rm{find}}\quad {\bf{k}},\quad {\rm{\min }}\quad {\tilde{\alpha }}_{ij}({\bf{k}})\\ {\rm{where}}\quad {\bf{k}}=({\sigma }_{\varphi },{\mu }_{\varphi },{\sigma }_{\theta },{\mu }_{\theta },{\sigma }_{\psi },{\mu }_{\psi },{v}_{f})\\ {\rm{s}}.{\rm{t}}\mathrm{.,}\quad 0 < {v}_{f} < 1\quad {\rm{and}}\\ {\rm{s}}.{\rm{t}}\mathrm{.,}\quad 0.5\,rad\mathrm{.}\le ({\sigma }_{\varphi },{\sigma }_{\theta },{\sigma }_{\psi })\le 5\,rad\mathrm{.}\quad {\rm{and}}\\ {\rm{s}}.{\rm{t}}\mathrm{.,}\quad 0\,rad\mathrm{.}\le ({\mu }_{\varphi },{\mu }_{\theta },{\mu }_{\psi })\le \pi /2\,rad.\end{array}\}$$

**Figure 3:**
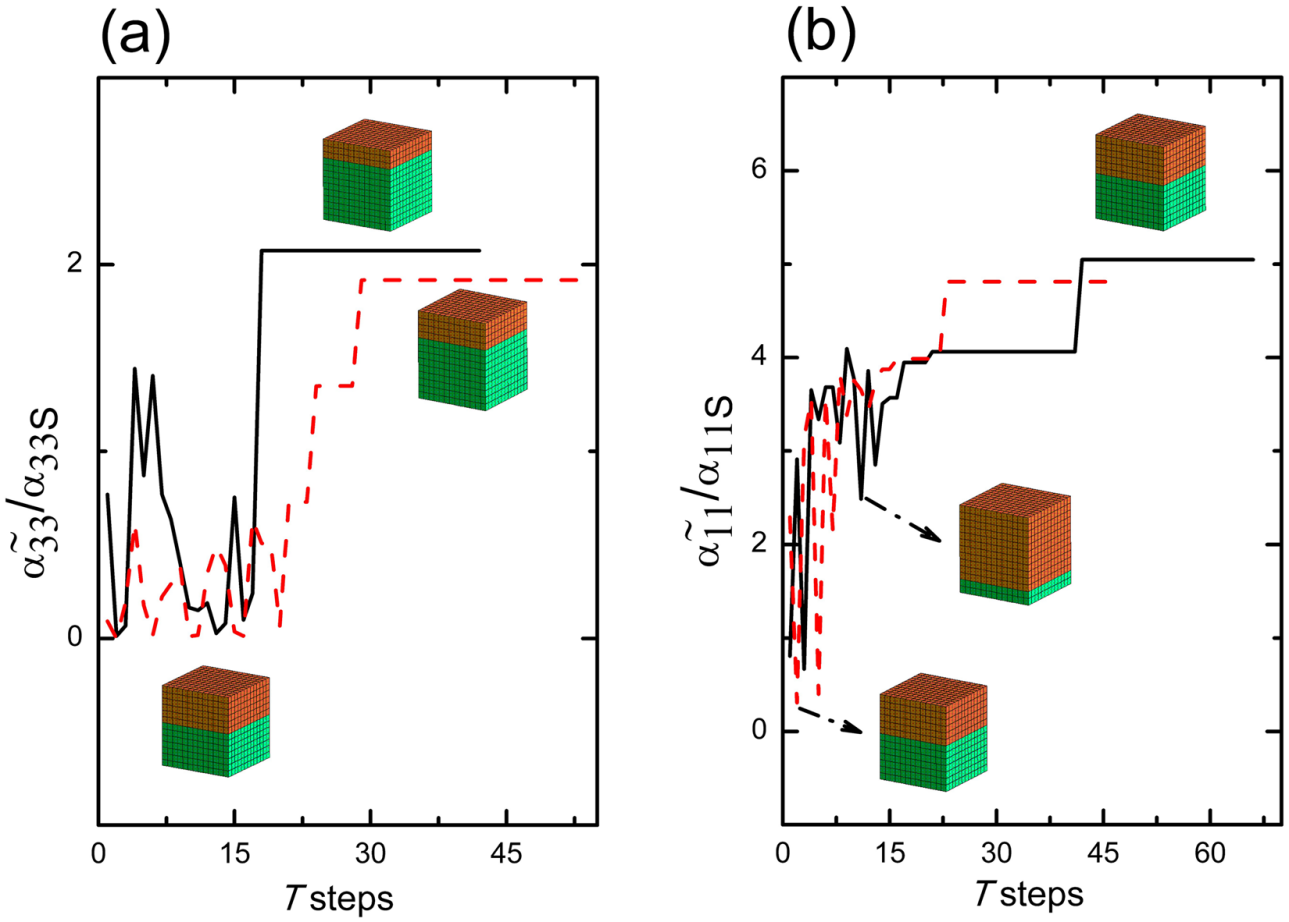
Convergence of the normalized ME coupling coefficients, (**a**) ${\tilde{\alpha }}_{33}$ and (**b**) ${\tilde{\alpha }}_{11}$ of the polycrystal BTO–ceramic CFO laminate for two initial guesses each. Here the ${\tilde{\alpha }}_{ijS}$ are the effective ME coupling of the ceramic BTO–CFO laminate.

Here the design variables are the standard deviation *σ* and mean *μ* of the normal distribution of grain orientations of the FE phase (BTO) of the ME laminate. We have assumed a normal distribution of orientation of the grains in Eq. 3 and hence we have ascribed a standard deviation *σ*_*χ*_ about a mean *μ*_*χ*_ where *χ* ≡ {*ϕ*, *θ*, *ψ*} the set of Euler angles. Thus the solution in Table 2 carries the optimal values obtained for three (*σ*_*χ*_, *μ*_*χ*_) pairs. Figure 3(a,b) show the results of this optimization. The MSs shown inset provide only the volume fraction of the constituent BTO and CFO phases of the initial guesses and the solution. In bulk BTO–CFO type eutectic composite samples, the volume fraction at which the maximum ME coupling is realized comes below the value obtained in our study (Table 2), except the one reported in ref.^42^ where it is congruent with our results. For instance, in BaTiO_3_-Ni(Co, Mn)Fe_2_O_4_, the volume fraction of BTO was obtained as *v*_*f*_ = 0.4 (ref.^43^) while in another BTO–CFO eutectic composite it was found to be *v*_*f*_ = 0.6 (ref.^44^). Analytical results on *α* show an increase in the value until *v*_*f*_ of BTO turns 50% and then remains constant^4^. The ME coupling relies on many factors, such as the stoichiometry, lack of texture, disparity of the character of the composite due to chemical modifications, porosity etc^2,7^.

**Table 2:** The normalized optimal values of effective ${\tilde{\alpha }}_{33}$ and ${\tilde{\alpha }}_{11}$ of the polycrystal BTO–ceramic CFO composite laminate and the corresponding solutions v_*f*_ and (*μ*_*θ*_, *σ*_*θ*_, *μ*_*ϕ*_, *σ*_*ϕ*_, *μ*_*ψ*_, *σ*_*ψ*_) (in radians) of the FE (BTO) component.

Objective $\widetilde{{{\boldsymbol{\alpha }}}_{{\boldsymbol{ij}}}}$	Case	Solution-($({{\boldsymbol{\mu }}}_{{\boldsymbol{\varphi }}},{{\boldsymbol{\sigma }}}_{{\boldsymbol{\varphi }}})\parallel ({{\boldsymbol{\mu }}}_{{\boldsymbol{\theta }}},{{\boldsymbol{\sigma }}}_{{\boldsymbol{\theta }}})\parallel ({{\boldsymbol{\mu }}}_{{\boldsymbol{\psi }}},{{\boldsymbol{\sigma }}}_{{\boldsymbol{\psi }}})$	*v*_*f*_	$\widetilde{{{\boldsymbol{\alpha }}}_{{\boldsymbol{ij}}}}/\widetilde{{{\boldsymbol{\alpha }}}_{{\boldsymbol{ijS}}}}$ (Normalized)
$\widetilde{{\alpha }_{33}}$	1	$\mathrm{(1.57},\mathrm{2.8)}\parallel \mathrm{(0.17},\mathrm{0.5)}\parallel \mathrm{(0.52},\mathrm{2.3)}$	0.77	2.07
	2	$\mathrm{(1.13},\mathrm{1.8)}\parallel \mathrm{(0.17},\mathrm{0.5)}\parallel \mathrm{(1.48},\mathrm{1.1)}$	0.69	1.91
	Experiment^a^	—	0.68	—
$\widetilde{{\alpha }_{11}}$	1	$\mathrm{(0.87},\mathrm{0.8)}\parallel \mathrm{(0.26},\mathrm{0.5)}\parallel \mathrm{(0.09},\mathrm{0.6)}$	0.54	5.04
	2	$\mathrm{(0.52},\mathrm{1.4)}\parallel \mathrm{(0.09},\mathrm{0.5)}\parallel \mathrm{(1.05},\mathrm{2.4)}$	0.54	4.81

^a^Pelletized bulk samples of the sintered and poled BaTiO_3_–modified spinel CoFe_2_O_4_ composite from ref.^42^.

**Figure 4:**
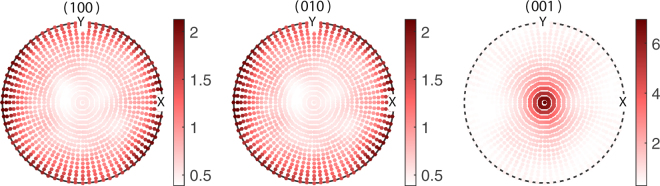
Pole figures of the FE BTO phase for the initial guess of ${\tilde{\alpha }}_{33}$ optimization of the polycrystal BTO–ceramic CFO laminate.

**Figure 5:**
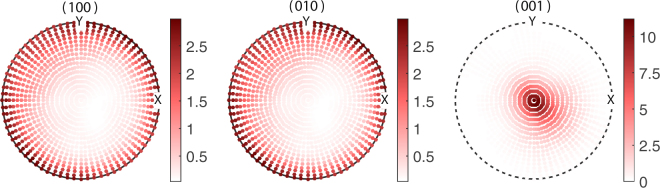
Pole figures of the BTO phase for the solution of ${\tilde{\alpha }}_{33}$ optimization of the polycrystal BTO–ceramic CFO laminate.

**Figure 6:**
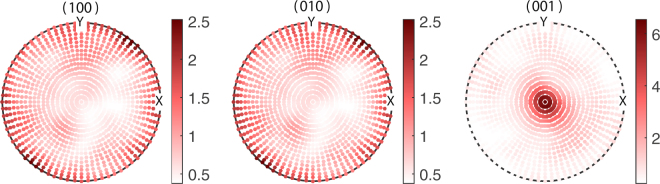
Pole figures of polycrystal BTO with 338 grains for the initial guess of ${\tilde{\alpha }}_{11}$ optimization of the polycrystal BTO–ceramic CFO laminate.

**Figure 7:**
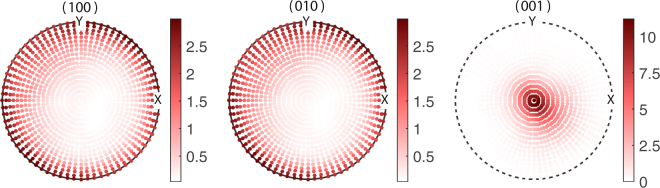
Pole figures of polycrystal BTO with 1183 grains for the solution of ${\tilde{\alpha }}_{11}$ optimization of the polycrystal BTO–ceramic CFO laminate.

The stereographic projections, the (hkl) pole figures of BTO essentially plots the poles projected by the [hkl] directions on the reference sphere constructed with the local coordinate system as its radii^45^. Figure 4 gives the pole figures of the planes {100}, {001} and {001} of the grains of BTO used in the starting configuration of the ${\tilde{\alpha }}_{33}$ optimization. The orientations of BTO were generated based on the values $({\mu }_{\varphi },{\sigma }_{\varphi })\parallel ({\mu }_{\theta },{\sigma }_{\theta })\parallel ({\mu }_{\psi },{\sigma }_{\psi })=\mathrm{(0},\mathrm{1)}\parallel (0,\mathrm{1)}\parallel \mathrm{(0},\mathrm{1)}$ with 1183 grains for the starting point of the optimization algorithm. Figure 4 represents some texture as we can see nonuniform accumulation of poles in all three cases, yet it creates a normalized ME coupling ${\tilde{\alpha }}_{33}/{\tilde{\alpha }}_{33S}=0.77$. However, we can see a larger concentration of poles in the solution pole figure of Fig. 5 especially in the (001) pole figure. Unlike the (001) and (010) pole figures, the (001) pole figure in Fig. 5 display maximum clustering of poles around the centre. Here the number of grains in MS become 1690 at optimum. This signifies the fact that majority of [001] axes, the spontaneous polarization direction of BTO, are oriented parallel to the *y*_3_ direction of the lamina. This implies that in order to achieve the enhanced out of plane coupling, one needs to have a texture which favours the alignment of maximum number of grains (or rather its spontaneous polarizations) towards the *y*_3_ axis. Moreover, it is seen that majority of poles in the (100) and (010) pole figures in Fig. 5 are concentrated along the circumference. This indicates that in the optimum configuration where the ME coupling ${\tilde{\alpha }}_{33}$ is maximized, the ab-planes of the majority of BTO crystallites lies parallel to the laminar plane while obviously the c-axes lie out of plane of the laminar plane. i.e., majority of the BTO domains constituting the FE phase are c-domains where the c-axes of the unit cells lie parallel to the laminate normal. Nanocomposite ME films were shown to exhibit strong ME coupling consequent to preferential orientation of the FE (PZT) layer favoring the formation of c-domains is shown in the present study^10,46^. The pole plots for the optimization of ${\tilde{\alpha }}_{11}$ are shown in Figs 6 and 7. The initial guesses used for pole figures (Fig. 6) are $({\mu }_{\varphi }=\pi /4,{\sigma }_{\varphi }=\mathrm{1)}\parallel ({\mu }_{\theta }=\pi /4,{\sigma }_{\theta }=\mathrm{1)}\parallel ({\mu }_{\psi }=\pi /4,{\sigma }_{\psi }=\mathrm{1)}$. Here we can conclude from the third picture of Fig. 7 as the texture is [001]-oriented. Thus, if we introduce proper texture you can get enhancements of ME coupling to the tune of ${\tilde{\alpha }}_{11}\sim 5$ times and ${\tilde{\alpha }}_{33}\sim 2$ times that of the bulk composite (Table 2).

### ME voltage coefficient *α*_*E*_

Another parameter of importance is the overall ME voltage coefficient ${\tilde{\alpha }}_{\mathrm{E}}=\delta E/\delta H$ of the laminate. Generally, ${\tilde{\alpha }}_{\mathrm{E}}$ is determined when the sample is subjected to a bias field *H* and an AC field *δH* by measuring the electric field *δE*^12^. The strong ME coupling is expected in a layered structure primarily due to low leakage currents and ease of poling to align the electric dipoles and thereby strengthen the piezoelectric effect of the ferroelectric phase of the composite^27^. For the out-of-plane ME voltage coefficient ${\tilde{\alpha }}_{E33}={\tilde{\alpha }}_{33}/{\tilde{\kappa }}_{33}$ at the optimal configuration^8^, we get a value of ${\tilde{\alpha }}_{E33}\approx 193$ V/cmOe for single crystal BTO-ceramic CFO laminate. Here, ${\tilde{\alpha }}_{33}$ = −1.546 × 10^−9^ Ns/VC and ${\tilde{\kappa }}_{33}$ = −0.637 × 10^−11^ F/m at the optimum obtained in the study. While for ceramic BTO–CFO laminate the optimal value of ${\tilde{\alpha }}_{E33}$ obtained is ${\tilde{\alpha }}_{E33}\approx 143$ V/cmOe. (Here the ${\tilde{\alpha }}_{33}$ = −0.574 × 10^−9^ Ns/VC and ${\tilde{\kappa }}_{33}$ = −0.319 × 10^−11^ F/m at the optimum. Epitaxially grown CoFe_2_O_4_–BaTiO_3_ heterostructure on the 001–SrTiO_3_ substrate via pulsed laser deposition displayed an out-of-plane ME coefficient of 104 mV/cmOe, which is orders of magnitude lower than the present value, however^35^. Ren *et al*.^47^ obtained value up to 2.54 V/cmOe at 160 kHz, for a particulate composite of BTO–CFO synthesized by a one-pot process. The experimental values are generally lower in the case of particulate composites compared to laminates, due to elimination of leakage problem^10^. We assume a perfect interface between the laminae which is not the ideal case in experiments^8^. In the case of heteroepitaxial thin film configurations such as in ref.^35^, the clamping due to stiff substrate would essentially restrict the ME coupling response^10^. Besides this, the discrepancy between the theory and the experiment is due to a host of other factors such as; defects of the sample chiefly due to interphase diffusion of the constituent atoms and the consequent deterioration of piezoelectricity and magnetostriction of the constituent phases resulted in poor strain transfer across phases, porosity, large thermal expansion mismatch and the subsequent formation of microcracks, demagnetization, leakage currents etc.^7,8^.

Some multiferroic laminate composites of the transversely poled Poly(vinylidene fluoride–co–hexafluoropropylene-P(VDF-HFP) and the iron-based Metglas fabricated by Jin *et al*.^24^ shows *α*_*E*33_ values as high as 320 V/cmOe. (001)-oriented PMN–PT crystal laminated with (211)-grain oriented Terfenol–D was found to yield ME voltage coefficient *α*_*E*33_ = 30.8 V/cmOe at a resonance frequency of ~78 kHz^25^. The ME response of a five layer Metglas/Terfenol-D/PMN-PZT single crystal/Terfenol-D/Metglas laminate at 1 kHz was found to be 5 V/cmOe^48^. These values fall in the range of and resembles the values obtained for the ME laminates obtained in the present study. Strong ME effects in a variety of ferrite based composites, including thin film piezoelectrics on nickel zinc ferrite, dense films of nickel ferrite and a piezoelectric deposited by aerosol deposition, in multiferroic magnetostrictive/piezoelectric composite bimorph structures are reported recently^9,25–27^. Recent advances in manufacturing single crystal oriented multilayer films through heteroepitaxial growth and multilayering is found to enhance the magnetoelectric coupling^6^. In epitaxial growth of thin films, an oriented crystalline film is deposited onto a crystalline substrate, while in this study a polycrystalline ferromagnetic layer bonded to a crystalline ferroelectric layer. A recent report^49^, however presents an approach namely combinatorial substrate epitaxy (CSE) relies on the creation of polycrystalline substrates and provide access to a wide range of materials and numerous orientations in a single pass. Studies in this direction would plausibly promote experiments aimed at realizing optimal multiferroic material geometries where the ME coupling is maximized as is manifested in the present study.

In summary, a computational framework for the material design and pole figure analysis of ME composites identified a range of grain orientation/distribution of the FE phase in ME composite wherein the ME coupling can be enhanced manifold is identified. The single crystal phase of BTO is found to be ideal for the maximum ME coupling configuration compared to the polycrystalline phase. A systematic increase in interfacial shear strains is identified with the alignment of the *c*-axis of the BaTiO_3_ single crystal phase of the composite along the global *z*–axis. Yet, the data pertaining to the ceramic BTO-CFO demonstrates unique solutions for the extreme coupling comparable to the single crystalline phase which can provide the manufacturers with more degrees of freedom. The insight obtained from the optimization has the potential to advance the design and upscaling of complex ME composite configurations with superior coupling. Further studies on laminates consisting of relaxor ferroelectrics, which possesses larger piezoelectricity, and ferromagnets with high piezomagnetic coupling such as ferrites, transition metals and rare-earth alloys could inaugurate new possibilities in technological applications demanding larger ME coupling.

See Supplementary material for the details on the multiferroic homogenization, convergence analysis for representative volume element and the optimization algorithm.

## Electronic supplementary material

Supplementary Material

## References

[1] Ortega N, Kumar A, Scott JF, Katiyar RS (2015). Multifunctional magnetoelectric materials for device applications. J Phys Condens Matter.

[2] Martin LW, Ramesh R (2012). Multiferroic and magnetoelectric heterostructures. Acta Mater.

[3] Cai K (2017). Electric field control of deterministic current-induced magnetization switching in a hybrid ferromagnetic/ferroelectric structure. Nat Mater.

[4] Avellaneda M, Harshe G (1994). Magnetoelectric effect in piezoelectric magnetostrictive multilayer (2–2) composites. J. Intell. Mater. Syst. Struct..

[5] Huang C-Y (2015). Imaging magnetic and ferroelectric domains and interfacialspins in magnetoelectric La0.7Sr0.3 MnO3/PbZr0.2Ti0.8O3 heterostructures. J Phys Condens Matter.

[6] Liu J, Zhang Y, Lin Y, Nan CW (2009). Magnetoelectric coupling inBaTiO3/(NiFe2O4/BaTiO3)_*n*_ (n = 1, 2, 3, 4) multilayered thin films. J Appl Phys.

[7] Fiebig M (2005). Revival of the magnetoelectric effect. J. Phys. D: Appl. Phys..

[8] Nan CW, Bichurin MI, Dong S, Viehland D, Srinivasan G (2008). Multiferroic magnetoelectric composites: Historical perspective, status, and future directions. J. Appl. Phys.

[9] Zhai J, Xing Z, Dong S, Li J, Viehland D (2008). Magnetoelectric laminate composites: An overview. J Amer Ceram Soc.

[10] Ma J, Hu J, Li Z, Nan C-W (2011). Recent progress in multiferroic magnetoelectric composites: from bulk to thin films. Adv. Mater..

[11] Zhai J, Cai N, Shi Z, Lin Y, Nan C-W (2004). Coupled magnetodielectric properties of laminated PbZr0.53Ti0.47O3/NiFe2O4 ceramics. J Appl Phys.

[12] Srinivasan G (2010). Magnetoelectric composites. Annu. Rev. Mater. Res..

[13] Labusch, M., Schröder, J. & Keip, M.-A. AnFE^2^-scheme for magneto-electro-mechanically coupled boundary value problems. In *Ferroic Functional Materials*: *Experiment*, *Modeling and Simulation*, edited by Schröder, Jörg & Lupascu, Doru C. pp. 227–262 (Springer International Publishing, Cham, Switzerland, 2018).

[14] Nan C-W, Li M, Huang JH (2001). Calculations of giant magnetoelectric effects in ferroic composites of rare–earth-iron alloys and ferroelectric polymers. Phys. Rev. B.

[15] Bichurin MI, Petrov VM, Srinivasan G (2003). Theory oflow-frequency magnetoelectric coupling in magnetostrictive-piezoelectricbilayers. Phys. Rev. B.

[16] Ray, M. C. Enhanced magnetoelectric effect in multiferroic composite beams due to flexoelectricity and transverse deformations. *Int J Mech Mater Des*, 10.1007/s10999-017-9380-7 (2017).

[17] Harshe G, Dougherty JO, Newnham RE (1993). Theoretical modelling of multilayer magnetoelectric composites. Int. J. Appl. Electromagn. Mater..

[18] Ce-Wen N (1994). Magnetoelectric effect in composites of piezoelectric and piezomagnetic phases. Phys. Rev. B.

[19] Bravo-Castillero J, Rodríguez-Ramos R, Mechkour H, Otero JA, Sabina FJ (2008). Homogenization of magneto–electro–elastic multilaminated materials. Q. J. Mechanics Appl. Math.

[20] Li JY, Dunn ML (1998). Anisotropic coupled-field inclusion and inhomogeneity problems. Phil. Mag. A.

[21] Benvensite Y (1995). Magnetoelectric effect in fibrous composites with piezoelectric and piezomagnetic phases. Phys. Rev. B.

[22] Dong S, Li J-F, Viehland D (2003). Longitudinal and transverse magnetoelectric voltage coefficients of magnetostrictive/piezoelectric laminate composite: theory. IEEE T Ultrason Ferr.

[23] Zhang JX (2007). Phase-field model for epitaxial ferroelectric and magnetic nanocomposite thin films. Appl Phys Lett.

[24] Jin J (2014). Multiferroic polymer laminate composites exhibiting high magnetoelectric response induced by hydrogen-bonding interactions. Adv Funct Mater.

[25] Dong S, Zhai J, Bai F, Li J-F, Viehland D (2005). Push-pull mode magnetostrictive-piezoelectric laminate composite with an enhanced magnetoelectric voltage coefficient. Appl Phys Lett.

[26] Lu SG (2011). Large magnetoelectric coupling coefficient in poly(vinylidene fluoride-hexafluoropropylene)/metglas laminates. J Appl Phys.

[27] Srinivasan, G. Layered multiferroic composites. In *Composite Magnetoelectrics*, Woodhead Publishing Series in Electronic and Optical Materials, edited by Srinivasan, Gopalan, Priya, Shashank & Sun, Nian X. pp. 55–70 (Woodhead Publishing, 2015).

[28] The lattice parameter of CFO is *a* = *8*.*38* Å, and that of BTO are *a* = *b* = *3*.*99* Å and *c* = *4*.*04* Å. The latticemismatch f is defined by *f* = *2*(*a*_*f*_*− a*_*s*_)/*a*_*s*_*~* (*a*_*s*_*− a*_*f*_)/*a*_*f*_, where af and as are the lattice parametersof the film (in this case BTO) and substrate (in this case CFO) respectively.

[29] Kirkpatrick S, Gelatt CD, Vecchi MP (1983). Optimization by Simulated Annealing. Science.

[30] Metropolis N, Rosenbluth A, Rosenbluth M, Teller A, Teller E (1953). Equation of state calculations by fast computing machines. J. Chem. Phys..

[31] Zheng H, Kreisel J, Chu Y-H, Ramesh R, Salamanca-Riba L (2007). Heteroepitaxially enhanced magnetic anisotropy in BaTiO3-CoFe2O4 nanostructures. Appl. Phys. Lett..

[32] Martin LW, Rappe AM (2016). Thin-film ferroelectric materials and their applications. Nat Rev Mater.

[33] Jayachandran KP, Guedes JM, Rodrigues HC (2014). A generic homogenization model for magnetoelectric multiferroics. J Intel Mat Syst Str.

[34] Fiebig M, Lottermoser T, Meier D, Trassin M (2016). The evolution of multiferroics. Nat. Rev. Mater..

[35] Zhang Y, Deng C, Ma J, Lin Y, Nan C-W (2008). Enhancement in magnetoelectric response in CoFe_2_O_4_–BaTiO_3_ heterostructure. Appl Phys Lett.

[36] Zheng H (2004). Multiferroic batio_3_-cofe_2_o_4_ nanostructures. Science.

[37] Yang P (2009). Magnetoelectricity in laminate composites of Terfenol-D and 0.52 Pb(Fe 1/2 Nb1/2)O3-0.48PbTiO3 with different orientations. J Phys D: Appl Phys.

[38] Eerenstein W, Mathur ND, Scott JF (2006). Multiferroic and magnetoelectric materials. Nature (London).

[39] Nye JF (1985). Physical properties of crystals: their representation by tensors and matrices.

[40] Cheng Z (2014). “Interface strain-induced multiferroicity in a SmFeO3 film. ACS Appl. Mater. Interfaces.

[41] Wang Y, Hu J, Lin Y, Nan C-W (2010). Multiferroic magnetoelectric composite nanostructures. NPG Asia Mater.

[42] Mazumder S, Battacharyya GS (2003). Magnetoelectric behavior in *in situ* grown piezoelectric and piezomagnetic composite-phase system. Mater Res Bull.

[43] van den Boomgaard J, Born RAJ (1978). A sintered magnetoelectric composite material BaTiO3-Ni(Co, Mn)Fe2O4. J. Mater. Sci..

[44] Van Run AMJG, Terrell DR, Scholing JH (1974). An *in situ* grown eutectic magnetoelectric composite material. J. Mater. Sci..

[45] Hielscher R, Schaeben H (2008). A novel pole figure in version method: specification of the *MTEX* algorithm. J Appl Cryst.

[46] He H-C, Ma J, Wang J, Nan C-W (2008). Orientation-dependent multiferroic properties in Pb(Zr0.52Ti0.48)O3-CoFe2O4 nanocomposite thin films derived by a sol-gel processing. J Appl Phys.

[47] Ren SQ (2005). BaTiO3/CoFe2O4 particulate composites with large high frequency magnetoelectric response. J Mater Sci.

[48] Park C-S, Cho K-H, Arat MA, Evey J, Priya S (2010). High magneticfield sensitivity in Pb(Zr,Ti)O3-Pb(Mg1/3Nb2/3)O3 singlecrystal/Terfenol-D/Metglas magnetoelectric laminate composites. J Appl Phys.

[49] Wittkamper J (2017). Competitive growth of scrutinyite(*α*-PbO2) and rutile polymorphs of sno2 on all orientations of columbite conb2o6 substrates. Cryst Growth Des.

