# Supplementary material for: Solutions for maximum coupling in multiferroic magnetoelectric composites by material design
Source: Sci Rep. 2018 Mar 20;8:4866. doi: 10.1038/s41598-018-22964-9 (PMC5861126; doi:10.1038/s41598-018-22964-9)
Supplement: Supplementary file 1 — Supplementary Material [file 41598_2018_22964_MOESM1_ESM.pdf]

Supplementary Material to
Solutions for maximum coupling in multiferroic magnetoelectric composites by material design

K.P. Jayachandran,* J.M. Guedes, and H.C. Rodrigues
IDMEC, Instituto Superior Técnico, University of Lisbon, Av. Rovisco Pais, 1049-001 Lisbon , Portugal

I. MULTIFERROIC HOMOGENIZATION

We consider a magnetoelectric multiferroic body occupying a volume Ω and assume that the material properties change periodically and the period is characterized by the dimension of an elementary cell Y of the body. A hierarchical schematic diagram of a laminate magnetoelectric considered in the present study is shown in Figure S1. Here we can see the various coordinates involved in the study alongwith the schematics of the macroscopic structure and microstructure. The ferroelectric layer of the laminate is composed of polycrystalline $BaTiO_3$. $BaTiO_3$ is settled in tetragonal perovskite ABO_3 structure at room temperature[1]. This is shown schematically in the Figure S1 alongwith the Euler angle coordinate system in which the orientation of the crystallographic grains of $BaTiO_3$ is characterized. It is seen from the Euler angles $(\phi, , \theta, \psi)$ depicted in Figure S1 that it measure the rotation of the crystallographic coordinates (x, y, z) with reference to the microstructure coordinates (y_1, y_2, y_3). The bottom layer of the laminate is composed of the ferromagnetic material $CoFe_2O_4$ which is treated in this study without assigning any rotation whatsoever. The general homogenization method applied to magnetoelectric composite is based upon assumptions of periodic boundary conditions on the microstructure and the separation of the microstructure scale through asymptotic expansion.

II. REPRESENTATIVE VOLUME ELEMENT

In the present study, the magnetoelectric composite microstructure is discretized using finite elements (FE). There would arise a conflicting situation with regard to the choice of a meaningful microstructure or representative volume element (RVE) to be used for the analysis. The RVE should be a meaningful representative piece of the real magnetoelectric polycrystalline material so that the inhomogeneities will be reflected in it completely. Yet, its size should be small enough to be considered as a volume element of the continuum model. Meanwhile, it should be sufficiently large to be statistically representative of the polycrystal. The latter requirement will ensure the robustness of the FE solution as well as the randomness of the grain distribution but fails to uphold the compactness of the RVE. Moreover, the number of grains constituting the microstructure should be big enough to surpass the statistical fluctuations of the significant physical parameters characterizing the magnetoelectric phenomena. Hence, in order to arrive at a meaningful representative volume element, we have performed numerical simulations by varying the number of finite elements, N_E. This would be equivalent to varying the number of crystallographic grains to be added to the RVE since in the present study one grain corresponds to one finite element.

We have started with a microstructure discretized by 216 ($\equiv 6 \times 6 \times 6$ mesh) finite elements which correspond to as much number of grains. The laminate composite for this analysis is composed of equal volume of ferroelectric $BaTiO_3$ (BTO) and ferromagnetic $CoFe_2O_4$ (CFO) layers. A normal distribution of $N_E/2$ number of orientations are generated (with mean μ=0 and standard deviation σ=1) and picked up randomly to assign orientations to the grains in the RVE. Here we must notice that the orientation distribution is applied solely to ferroelectric phase and not to the ferromagnetic phase. The homogenized magneto–electromechanical moduli are calculated for BTO–CFO composite using the parameters of monodomain single-crystal data of BTO from Ref. 1 and ceramic data of CFO from Ref. 2 using the homogenization model computationally implemented in FORTRAN. Similar computations are repeated with the addition of more number of grains to the microstructure until the it reaches $20 \times 20 \times 20$ mesh where the RVE contains 4000 grains of BTO. The results obtained for homogenized magnetoelectric coefficients $\widetilde{\alpha_{ij}}$ are plotted in Figure S2. Since the set of orientations characterized by the Euler angles (ϕ, θ, ψ) are generated randomly using a random generator, it will be different each time the genera-

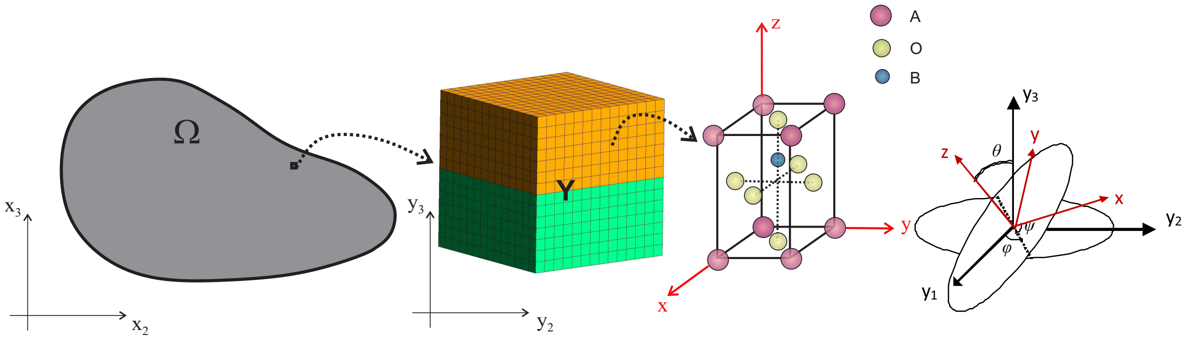

Figure S1. Hierarchical schematic diagram of the magnetoelectric multiferroic system showing various system of coordinates used in the present study. The macroscopic body Ω is a laminate with the microstructure Y wherein the top layer is the ferroelectric material possessing a perovskite ABO_3 structure such as $BaTiO_3$ and the bottom layer is a ferromagnet such as $CoFe_2O_4$.

tor is invoked. We generated three sets of (ϕ, θ, ψ) at standard deviation $\sigma = 1$ and mean μ=0 for each number of grains used in the simulation. We took the mean value of the $\widetilde{\alpha_{ij}}$ and is plotted in thick black line in Figure S2. The values obtained for $\widetilde{\alpha_{11}}$ and $\widetilde{\alpha_{33}}$ are slightly different each time. This scatter of values is shown as error bars (dark yellow vertical bars) and drawn by computing the standard deviation of values from the mean at each mesh size. Also, it can be seen the computational time in seconds taken in an Intel Core i7-3770K 3.5 GHz processor with 32 GB of RAM. Here we can see that the standard deviation of $\widetilde{\alpha_{ij}}$ keeps on decreasing with increase of mesh size while the computational time keeps on increasing drastically with mesh size. Hence we have to settle for a trade-off between computational cost and accuracy. As is seen from the figure, the accuracy we are gaining by discretizing the unit-cell is minimal above 1000 elements. Thus we chose a microstructure discretized by 2197 finite elements with a $13 \times 13 \times 13$ mesh would be used for the solution of the microscopic problems required for the evaluation of the homogenized magneto–electro–mechanical tensors. At around this mesh size, the standard deviation of $\widetilde{\alpha_{11}}$ is about ~ 0.04 and that of $\widetilde{\alpha_{33}}$ is about ~ 0.005.

III. OPTIMIZATION

Here the objective is to maximize the inplane magnetoelectric coupling coefficient $\widetilde{\alpha_{11}}$ and the out of plane magnetoelectric coefficient $\widetilde{\alpha_{33}}$ of a laminate magnetoelectric composite. The first problem is maximization of $\widetilde{\alpha_{11}}$ and $\widetilde{\alpha_{33}}$ in single crystalline BTO–ceramic CFO composite laminate. Here the design variables are the Euler angles (ϕ, θ, ψ) and the volume fraction v_f of the BTO. For the optimization procedure, a discrete set of angle values is chosen for each Euler angle, ranging from -180 degrees to $+180$ degrees in 5 degree increments, i.e.,

$$\{\phi, \theta, \psi\} \in \{-180^\circ, -175^\circ, \ldots, 175^\circ, 180^\circ\} \quad \text{(S1)}$$

Thus every angle set contains 73 elements and are introduced in radians. Moreover, the volume fraction vector contains elements as follows;

$$13 \times v_f = \{1, 2, \ldots, 11, 12\} \quad \text{(S2)}$$

Obviously, the design variable space contains a huge number of points derived from the permutations of the Euler angle and volume fraction vectors. Hence an exhaustive search is prohibitively expensive computationally and the problem warrants an algorithm such as the simulated annealing (SA) to accomplish the optimization. Moreover, simulated annealing is an efficient algorithm because of its inherent simplicity and ability to find the global optimum even if there are many design variables. The SA procedure differs from iterative improvement in that the procedure would not get stuck since transitions out of a local optimum are always possible at nonzero *temperature*[3]. Here the *temperature* is a control parameter in the SA algorithm that is expressed in the same unit as that of the objective function. The simulated annealing based in the Metropolis algorithm, draws analogy from the behavior of an ensemble of atoms that are cooled slowly from a melted state to the minimum energy (E) ground state.

The second optimization problem is the maximization of $\widetilde{\alpha_{ij}}$ in the polycrystalline BTO–ceramic CFO laminate. Here the design space is composed

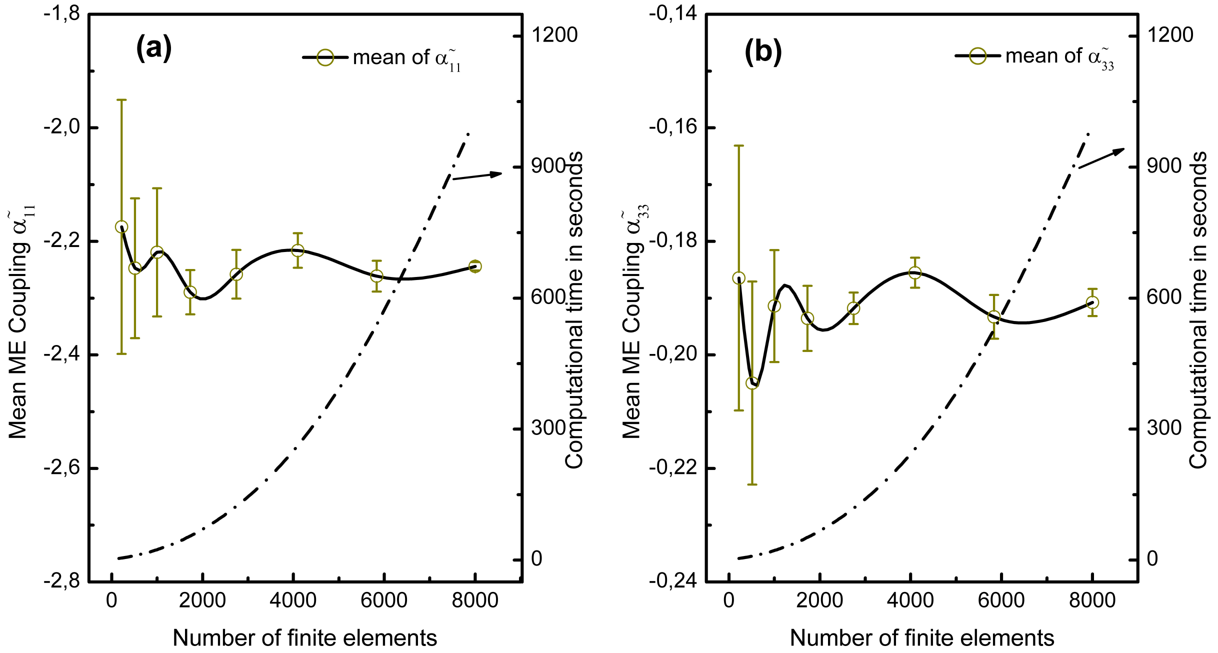

Figure S2. Convergence analysis for the optimization of the representative volume element (RVE) of the ceramic $BaTiO_3$(BTO)–$CoFe_2O_4$ (CFO)laminate. The variation of magnetoelectric (ME) (a) inplane coupling coefficient $\widetilde{\alpha}_{11}$ and (b) out of plane coupling coefficient $\widetilde{\alpha}_{33}$ (in the units of (10^{-9} Ns/VC) with the number of finite elements (grains) are shown. Here the error bars are drawn from the standard deviations of the $\widetilde{\alpha}_{ij}$ values obtained from three computations at each mesh size. The computational time for each homogenization run versus number of elements (the −. − .− curve) is also plotted.

of design variables $(\sigma_\phi, \mu_\phi, \sigma_\theta, \mu_\theta, \sigma_\psi, \mu_\psi, v_f)$. Here we assume that the polycrystalline BTO phase is composed of single crystalline grains whose orientations determined in Euler angels (ϕ, θ, ψ) and each of which fall in a normal distribution with mean μ_χ and satndard deviation σ_χ. Here χ stands for either of (ϕ, θ, ψ). Here too the design variable space is too big as in the previous optimization problem as follows;

$$\{\mu_\chi\} = \{0^\circ, 5^\circ, \ldots, 85^\circ, 90^\circ\} \qquad \text{(S3)}$$

are introduced in radians into the optimization program and

$$\{\sigma_\chi\} = \{0.5, 0.6, \ldots, \pi\} \qquad \text{(S4)}$$

The optimization algorithm would choose the design variables, *viz.*, σ, μ and v_f for each computation and according to it the set of Euler angles are generated from a normal distribution. These angles would be fed to each finite element of the BTO layer of the laminate. (The layer thickness is defined by the corresponding v_f in that optimization iteration.) The homogenized properties would be computed at this step and the optimization would compare this value with the successive value and would retain the same or discard according to the Metropolis algorithm. The algorithm surveys the design space almost uniformly during the optimization process as is seen from the Fig. S3.

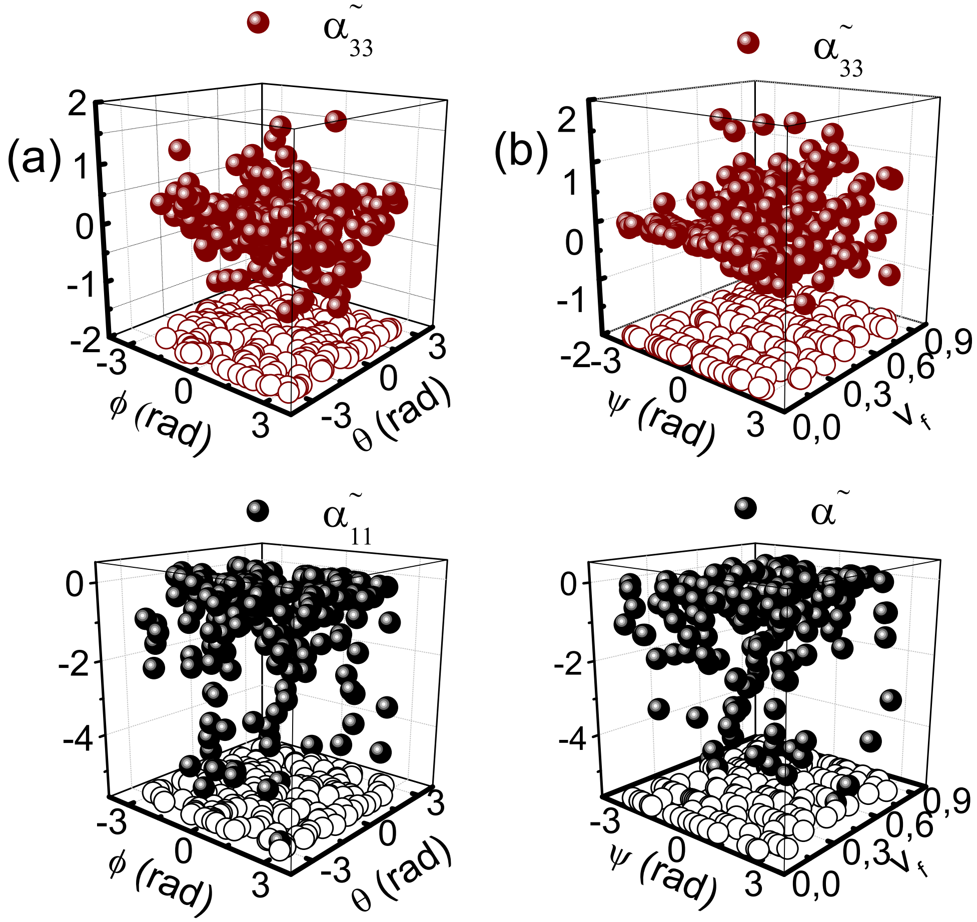

Figure S3. Variation in the objective functions $\widetilde{\alpha}_{33}$ and $\widetilde{\alpha}_{11}$, against the design variables chosen by the optimization algorithm at each ieteration of the single crystal BTO–ceramic CFO laminate composite. Each of the subplots shows $\widetilde{\alpha}_{ij}$ against the design variables, ϕ, θ, ψ and v_f (in radians) corresponding to each iteration. Here the open cirlces in the XY-plane of the figure shows the values of design variable pairs (ϕ, θ) and (ψ, v_f).

---

[*] kpjayachandran@gmail.com

[1] M. Zgonik, P. Bernasconi, M. Duelli, R. Schlesser, P. Gunter, M. H. Garrett, D. Rytz, Y. Zhu, and X. Wu, Phys. Rev. B **50**, 5941 (1994).

[2] E. Pan and P. R. Heyliger, J. Sound Vib. **252**, 429 (2002).

[3] S. Kirkpatrick, C. D. Gelatt, Jr., and M. P. Vecchi, Science **220**, 671 (1983).
